# His345 mutant of angiotensin-converting enzyme 2 (ACE2) remains enzymatically active against angiotensin II

**DOI:** 10.1073/pnas.2023648118

**Published:** 2021-04-08

**Authors:** Pan Liu, Xinfang Xie, Li Gao, Jing Jin

**Affiliations:** ^a^Feinberg Cardiovascular and Renal Research Institute, Department of Medicine-Nephrology and Hypertension, Northwestern University Feinberg School of Medicine, Chicago, IL 60611

Glasgow et al. (1) report their mutagenesis survey of ACE2 traps for treatment of severe acute respiratory syndrome coronavirus 2 (SARS-CoV-2). The virus invades human cells via host ACE2 receptor, and strategies aimed at disrupting this process are being explored. As compared to monoclonal antibodies and targeted vaccines that are prone to mutational escape of the virus, the ACE2 trap strategy has unique advantages and was shown to be efficacious in experimental and clinical tests (2, 3). Current studies, including Glasgow et al., focus on further improvements through alternative designs. These include extending in vivo half-life with an Fc tag (4, 5), increasing Spike-binding affinity via mutagenesis (1, 6), and inactivating ACE2 peptidase activity (1, 4). The Glasgow study combined all three approaches in their lead design (1).

**Fig. 1.**
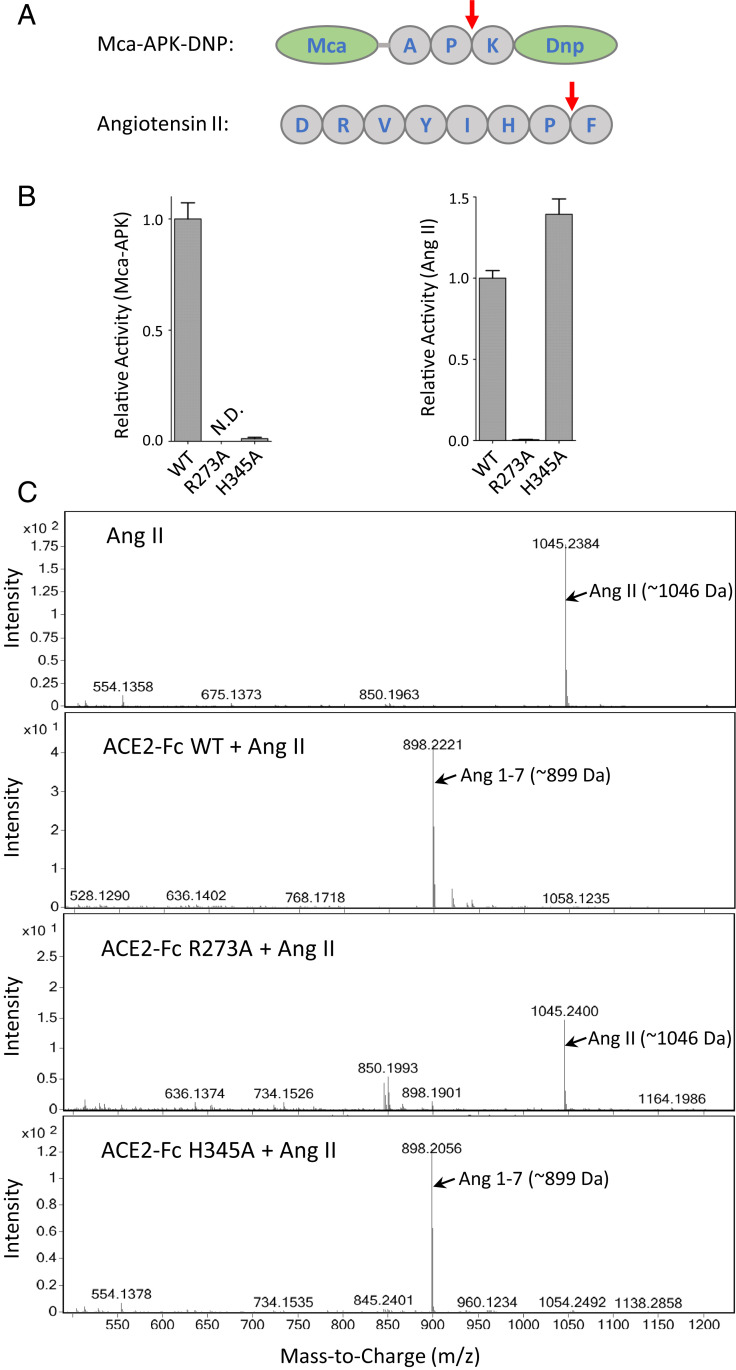
Substrate-dependent activities among ACE2 mutants. (*A*) Surrogate substrate Mca-APK-DNP is cleaved between P and K as compared to the P−F cleave site in Ang II by ACE2. (*B*) ACE2 activity assays were performed using either Mca-APK-DNP or Ang II as substrate, with individual variants of ACE2-Fc as the enzyme. H345A showed loss of activity toward the surrogate (*Left*; N.D. for not detected), in agreement with the findings by Glasgow et al. (1). However, ACE2-Fc enzymatic activity measured by Ang II showed different results, with H345A having full activity as compared to wild-type ACE2-Fc (*Right*). Meanwhile, R273A showed loss of activity against Ang II. (*C*) To further confirm the specificity of the reaction, we conducted mass spectrometry analysis of the peptide(s) generated from the reactions, using Ang II peptide as substrate, that further confirmed the loss of activity by R273A mutation, whereas H345A of ACE2 remained fully active.

The enzymatic inactivation strategy has two potential benefits: eliminating unwanted cardiovascular side effects attributable to dysregulation of vasoactive hormones, including angiotensin II (Ang II), and permitting high therapeutic doses without oversupplying ACE2 peptidase activity. It is unfortunate that many researchers, including Glasgow et al. (1), still choose surrogate substrate Mca-APK-DNP for measuring ACE2 activity, despite the apparent distinctions between Mca-APK-DNP and Ang II in sequence (Fig. 1*A*). Glasgow et al. examined two inactivating mutants of ACE2, namely H374N/H378N double mutations of the Zn^2+^-binding residues and H345L mutation at the catalytic center (7). The authors show loss of activity of both constructs against Mca-APK-DNP. Because the study discovers that H374N/H378N is unstable, they incorporate H345L in their lead candidate to have long action time (via Fc), better Spike binding (via affinity-enhanced mutation[s]), and being enzymatically inactivated (via H345L mutation).

In parallel to the Glasgow et al. (1) study, our group conducted a mutagenesis survey of ACE2-Fc toward inactivating its activity against physiologic substrates (8). We included mutations intended to disrupt either Zn^2+^ binding (via His374, His378, and Glu402) or substrate catalysis (via Glu145, Arg273, His345, Pro346, Asp368, and His505) with alanine substitutions of individual residues. We performed both Mca-APK-DNP assay and phenylalanine hydrolysis measurement using Ang II and Apelin-13 as substrates. Our results show a clear substrate-dependent inactivation among the mutants. Specifically, H345A, P346A, and H505A mutants show complete inactivation of ACE2-Fc against Mca-APK-DNP but intact catalysis against Ang II and Apelin-13. With respect to the H345 mutant (mutated to alanine in our study instead of to leucine as in Glasgow et al.), our results suggest the enzyme can still catalyze Ang II, which is a concern in therapeutic design for SARS-CoV-2.

To ascertain our findings, we repeated the assays and obtained similar results as before (Fig. 1*B*). In addition, we performed mass spectrometry to measure the generation of Ang(1–7) by ACE2-Fc R273A and H345A. The results confirm full activity of H345A toward Ang II (Fig. 1*C*), which argues against the conclusion by Glasgow et al. (1) with regard to the catalytic residue.
